# Identification and expression analysis of LEA gene family members in pepper (*Capsicum annuum* L.)

**DOI:** 10.1002/2211-5463.13718

**Published:** 2023-11-02

**Authors:** Yongyan Zhao, Yupeng Hao, Zeyu Dong, Wenchen Tang, Xueqiang Wang, Jun Li, Luyao Wang, Yan Hu, Lei Fang, Xueying Guan, Fenglin Gu, Ziji Liu, Zhiyuan Zhang

**Affiliations:** ^1^ Hainan Institute Zhejiang University Sanya China; ^2^ Zhejiang Provincial Key Laboratory of Crop Genetic Resources, Institute of Crop Science, Plant Precision Breeding Academy, College of Agriculture and Biotechnology Zhejiang University Hangzhou China; ^3^ Spice and Beverage Research Institute, Sanya Research Institute Chinese Academy of Tropical Agricultural Sciences/Hainan Key Laboratory for Biosafety Monitoring and Molecular Breeding in Off‐Season Reproduction Regions Sanya China; ^4^ Tropical Crops Genetic Resources Institute Chinese Academy of Tropical Agricultural Sciences/Key Laboratory of Crop Gene Resources and Germplasm Enhancement in Southern China, Ministry of Agriculture Haikou China

**Keywords:** cold stress, gene family, late embryogenesis abundant, placental development

## Abstract

Pepper (*Capsicum annuum* L.) is an economically important crop containing capsaicinoids in the seed and placenta, which has various culinary, medical, and industrial applications. Late embryogenesis abundant (LEA) proteins are a large group of hydrophilic proteins participating in the plant stress response and seed development. However, to date there have been no genome‐wide analyses of the LEA gene family in pepper. In the present study, 82 *LEA* genes were identified in the *C. annuum* genome and classified into nine subfamilies. Most *CaLEA* genes contain few introns (≤ 2) and are unevenly distributed across 10 chromosomes. Eight pairs of tandem duplication genes and two pairs of segmental duplication genes were identified in the LEA gene family; these duplicated genes were highly conserved and may have performed similar functions during evolution. Expression profile analysis indicated that *CaLEA* genes exhibited different tissue expression patterns, especially during embryonic development and stress response, particularly in cold stress. Three out of five *CaLEA* genes showed induced expression upon cold treatment. In summary, we have comprehensively reviewed the LEA gene family in pepper, offering a new perspective on the evolution of this family.

## Graphical abstract

Within the *Capsicum annuum* genome, there exist 82 LEA genes that group into nine different subfamilies, principally playing a role in placental development and the response to cold stress.

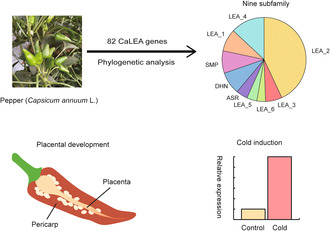

**Abbreviations**
ABA: abscisic acid
ASR: abscisic acid‐, stress‐, and ripening‐induced proteins
DHN: dehydrin
ET: ethylene
FPKM: the fragments per kilobase of exon model per million mapped reads
LEA: late embryogenesis abundant
MeJA: methyl jasmonate
MYA: million years ago
qRT‐PCR: quantitative reverse transcription polymerase chain reaction
SA: salicylic acid
SMP: seed maturation protein

The late embryogenesis abundant (LEA) protein is a highly hydrophilic glycine‐rich protein first identified 40 years ago in cotton (*Gossypium hirsutum*) seeds during late embryonic development [1]. Further studies have shown that LEA proteins are widely distributed in various species, including higher plants, bacteria, certain fungi, and invertebrates [2, 3, 4, 5]. In addition to the seeds, LEA proteins also exist in the leaves, stems, roots, and other plant tissues [6, 7, 8]. Many LEA proteins have been divided into several types based on different criteria in higher plants. Classification based on the similarity between amino acid sequences and conserved motifs available in the Pfam database is currently the most common approach [9, 10]. Based on these criteria, LEA proteins can be divided into nine groups: LEA_1–6, seed maturation protein (SMP), dehydrin (DHN), and abscisic acid‐, stress‐, and ripening‐induced proteins (ASR). ASR was recently identified as a novel LEA protein [10, 11]. Seed size is regulated by complex networks combining multiple developmental and environmental signals [12]. Late embryogenesis abundant proteins are instrumental in seed development in higher plants, and its expression can be considered as a clear indication of seed maturation [13, 14]. Some LEA proteins are crucial for seed longevity, desiccation tolerance, and endosperm quality control [15, 16, 17, 18]. For example, the weight, size, and fatty acid content of *Arabidopsis* seeds overexpressing *LuLEA1* are reduced [19].

Harmful environment can lead to an overall loss of approximately 70% of the yield for key agricultural crops, that is, an average yield of only approximately 30% of the genetic yield potential [20]. Most LEA proteins are highly hydrophilic and intrinsically disordered as they are abundant in charged amino acid residues [3, 6, 21]. Owing to these features, LEA proteins are relatively stable under heat and acid stress [22]. The induction of LEA proteins was considered to be a key component of the trophic desiccation tolerance strategy in resurrection plants [23, 24]. Recently, the functions of LEA proteins in abiotic stress tolerance have been studied. Drought, low temperature, salt stress, and other stressors can induce the expression of *LEA* genes, which also function under these conditions [13, 25, 26]. For instance, the expression of LEA proteins from *Gastrodia elata* in *Escherichia coli* can enhance tolerance to low‐temperature stress [27]. When overexpressing *OsLEA3‐2* in *Arabidopsis* and rice, the growth of transgenic plants was better than that of wild‐type plants under salt and drought conditions [28]. Additionally, the downregulation of LEA_4 subgroup genes results in sensitivity to water deficit in *Arabidopsis*, which may be related to the maintenance of cellular function by LEA proteins during water deficit [5, 7]. Furthermore, studies have indicated that LEA proteins perform vital functions under stress, acting as free radical scavengers under dry conditions [29, 30], preventing the inactivation of a variety of enzymes, such as lactate dehydrogenase, during water deficit [31, 32], and protecting proteins and membranes from adverse structural changes caused by dehydration [33].

So far, the genome‐wide identification and analysis of the LEA gene families have been conducted in several plant species with sequenced genomes, such as *Arabidopsis thaliana*, *Oryza sativa*, *Cucumis sativus* L., *Solanum lycopersicum*, *Camellia sinensis* (L.) O. Kuntze, *G. hirsutum*, and *Salvia miltiorrhiza* [9, 13, 25, 26, 34, 35, 36]. Pepper (*Capsicum annuum* L.), an important cash crop belonging to the Solanaceae family, is cultivated worldwide. Its fruits can be used not only as fresh vegetables and spices but also as raw materials for processing medicine and cosmetics [37]. Until now, studies on the function of *CaLEA* genes focused on responses to biotic and abiotic stresses. *CaLEA73* gene enhances drought and osmotic tolerance modulating transpiration rate in transgenic *A. thaliana* [38]. CaLEA6 protein plays a protective role in water deficit caused by dehydration and high salinity [39]. *CaLEA1* is involved in regulating ABA signaling, drought, and salt stress response [40]. The function of *CaDHN5* is related to salt and osmotic stress responses [41]. Although there have been a few studies on the function of *LEA* gene in pepper, there has not been a comprehensive and systematic identification of CaLEA gene family. In this study, 82 candidate LEA proteins were identified in the genome of *C. annuum* L. CM334, and their structures, evolution, chromosomal locations, and expression profiles in response to abiotic stresses were analyzed. These results provide a strong platform for identifying the biological functions of *CaLEA* in pepper plants.

## Materials and methods

### Identification of CaLEA genes in *C. annuum*

We downloaded the genome sequence of *C. annuum* from the *C. annuum* (cultivar CM334) genome database (http://peppergenome.snu.ac.kr) [37]. Hidden Markov Model profiles of *LEA* genes were obtained from the Pfam database and used to search the local genome database of *C. annuum*. All candidate genes were confirmed using the Pfam (http://pfam‐legacy.xfam.org/) [42] and NCBI Conserved Domain Search databases (https://www.ncbi.nlm.nih.gov/Structure/cdd/wrpsb.cgi). Eventually, all the LEA gene family members of pepper (*CaLEA*) were identified and named according to their positions on the chromosome. The ProtParam online tool (https://web.expasy.org/protparam/) was used [43] to estimate the following physical and chemical properties: grand average hydropathy (GRAVY), isoelectric point (pI), and molecular weight (MW).

### Phylogenetic analysis, gene structure and motif distribution and promoter analysis of CaLEA

To reveal the phylogenetic relationships of LEAs among *C. annuum* L, *A. thaliana*, *Solanum tuberosum*, and *S. lycopersicum*, multiple LEA protein sequences were aligned and a phylogenetic tree in mega 11.0 using the neighbor‐joining (NJ) method [44]. Group patterns were evaluated using bootstraps (1000 replicates) in the phylogenetic tree. tbtools software was used to identify exon–intron structures [45]. Conserved protein motifs were identified using meme (multiple expectation maximization for motif elicitation) (http://meme‐suite.org/tools/meme) [46], and the maximum number of different motifs was 20. Promoter sequences (2000 bp upstream of the translation start site) of *CaLEA* genes were extracted from the pepper genome dataset (http://peppergenome.snu.ac.kr). The PlantCARE database (http://bioinformatics.psb.ugent.be/webtools/plantcare/html/) was used to search for the cis‐acting elements.

### Gene duplication and evolutionary analysis

The *CaLEA* genes were mapped on the pepper chromosome based on the location information of *CaLEA* genes in the pepper database (http://peppergenome.snu.ac.kr) [37] and visualized using tbtools [45]. The segment and tandem of the putative duplication of the *CaLEA*s were constructed using mcscanx software [47], with the following testing parameters: *e* value of protein ratio less than 1e‐3, number of blast hits more than 10, and CPU for blastp was 2. The non‐synonymous substitution rate (*K*_a_) to the synonymous substitution rate (*K*_s_) was calculated using tbtools [45]. *K*_a_/*K*_s_ < 1, *K*_a_/*K*_s_ = 1, and *K*_a_/*K*_s_ > 1 represent the purifying selection effects, neutral selection, and positive (or diversifying) selection, respectively. We calculated divergence time using the formula *T* = *K*_s_/2*r*, where *r* is 1.5 × 10^−8^ synonymous substitutions per site per year [48].

### Expression pattern analysis based on the transcriptome data

The following transcriptome data were downloaded from the NCBI Sequence Read Archive (SRA) database (https://www.ncbi.nlm.nih.gov/sra) Reference comparisons were performed using hisat2 [49] software, and the fragments per kilobase of exon model per million mapped reads (FPKM) were calculated using stringtie [50] software. The fold change between the treatment group and the control group was calculated using the formula log_2_(FPKM(treatment) + 1)/(FPKM(control) + 1) to assess the changes following biotic and abiotic stress treatments. The resulting data were then imported into the r package called pheatmap to generate a heatmap.

### RNA isolation and quantitative reverse transcription polymerase chain reaction analysis

Pepper plants were grown under greenhouse conditions: 16 h light/8 h dark, 25–28 °C. When the pepper had 6 true leaves, the experimental group was cold‐treated in an incubator (16 h light/8 h dark, 10 °C), and the leaves were collected at 0, 3, 6, 12, 24, and 72 h after the treatment, immediately frozen, and stored in a −80 °C refrigerator. We used TIANGEN's polysaccharide‐ and polyphenolic‐rich RNAprep pure plant plus kit to extract total RNA from pepper leaves of *C. annuum* cv. CM334. Reverse transcription was performed using HiScript II Q RT SuperMix for qPCR (+gDNA wiper; R223‐01; Vazyme, Nanjing, China) following the manufacturer's instructions. We selected nine genes whose gene expression increased significantly after cold treatment in transcriptome analysis. The PCR was amplified using gene‐specific primers (Table S5) with ChamQ Universal SYBR qPCR Master Mix (Q711; Vazyme). To normalize the samples, the ubiquitin‐conjugating gene (*CaUbi3*) was used as an internal control. Each sample was analyzed three times, and the relative gene expression level was calculated using the $2^{-\Delta \Delta C_{T}}$ method.

### MicroRNA targeting analysis

Genomic sequences of 9 LEA members shown in Fig. 6 were submitted as candidates to predict of potential miRNAs, using the default parameters of psRNATarget online website (https://www.zhaolab.org/psRNATarget/). The uploaded miRNA sequences and the method of predictive analysis were previously reported [51, 52].

## Results

### Identification and characteristics of CaLEA genes in *C. annuum*

**Fig. 1:**
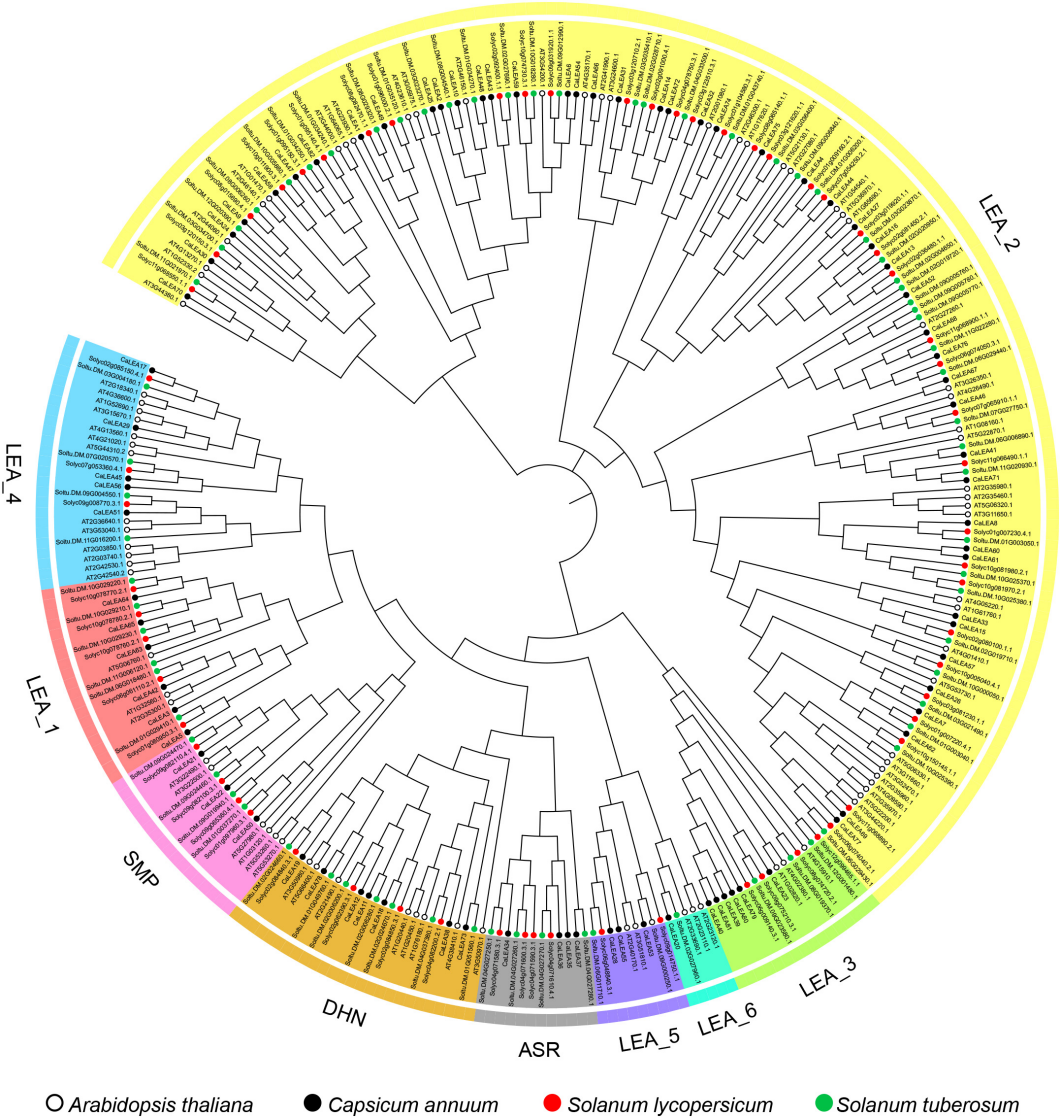
Phylogenetic relationships of *LEA* genes in pepper, tomato, potato, and *Arabidopsis*. The phylogenetic tree was constructed using mega 11.0, and the nine major groups are marked with differently colored backgrounds. Black, red, green, and white circles represent pepper, tomato, potato, and *Arabidopsis*, respectively.

We identified 82 *CaLEA* genes based on a Pfam ID search for *C. annuum* genome databases and a Pfam domain search for the CDD database using the publicly available genome sequence data of *C. annuum*. These *CaLEA*s were named *CaLEA1* to *CaLEA82* according to their order on chromosomes. To better understand the similarities, differences, and evolutionary relationships among *LEA* genes, phylogenetic analysis was performed using 302 LEA proteins from *A. thaliana*, *C. annuum*, *S. lycopersicum*, and *S. tuberosum* (Fig. 1). The results showed that these genes could be divided into nine groups: LEA_1–LEA_6, SMP, DHN, and ASR (Table S1). Based on phylogenetic analysis, we found that the largest group of LEA genes in pepper was LEA_2, which contained 47 members, whereas the smallest group was LEA_6, which had only one member. Additionally, groups LEA_1, LEA_3, LEA_4, LEA_5, DHN, SMP, and ASR contained 3–7 members.

Analysis of physiochemical properties revealed that the length of CaLEA proteins varied from 77 to 554 amino acids, and their molecular weights ranged from 8.56 to 57.82 kDa. The theoretical isoelectric points of the CaLEA proteins were between 4.26 and 10.51, of which 54 (66%) were considered basic (pI > 7), and 28 were considered acidic (pI < 7). Ninety percent of LEA_2 proteins and all LEA_3 proteins are basic, while other subgroups of proteins are mostly acidic. Moreover, analysis of the grand average of hydropathicity (GRAVY) index confirmed that except for the hydrophobic and hydrophilic proteins of LEA_2 subgroup, other subgroup proteins are hydrophilic (Table S1).

### Gene structure and conserved motifs of CaLEA genes

**Fig. 2:**
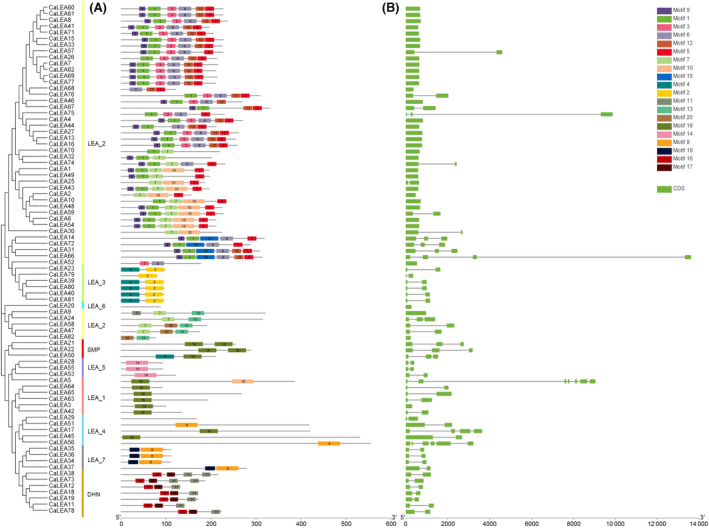
Phylogenetic tree, conserved motifs, and gene structures of *CaLEA* genes. (A) Conserved motif analysis of CaLEA proteins. Conserved motifs are numbered and indicated in colored boxes. Motif sequences are provided in Table S2. (B) Exon–intron structures of *CaLEA* genes. Green Bar: exons; black lines: introns.

Gene structure is an important factor in determining gene function. We analyzed the exon–intron structure and conserved motifs of *CaLEA* genes in pepper plants. Most *CaLEA* genes contained no introns or one intron, except for *CaLEA14*, *CaLEA21*, *CaLEA22*, *CaLEA24*, *CaLEA31*, *CaLEA50*, *CaLEA72*, and *CaLEA75* with two introns; *CaLEA17* and *CaLEA66* with three introns; *CaLEA56* with five introns; and *CaLEA5* with six introns. In general, the exon–intron structure of *CaLEA* genes in the same group was similar (Fig. 2B), which supports the classification of groups and their phylogenetic relationships.

To investigate the structural features of the CaLEA proteins, we used the MEME software to predict the conserved motifs (Table S2), and the maximum number of different motifs was 20 (Fig. 2A). Generally, most CaLEA protein sequences are highly variable, and different subgroups do not share a common conserved motif. From the motif analysis, it was clear that genes belonging to the same subfamily possessed several group‐specific conserved domains, and closer evolutionary relationships of genes were accompanied by more similar motif numbers. For instance, the LEA_5 and LEA_3 groups each had one specific motif, motif 14, and motif 2, respectively. Previous studies have reported that LEA proteins may require specific motifs for performing their specific functions to recognize and bind specific ligands. The DHN subfamily contains a lysine‐rich K segment (motif 11), which is an important structural basis for hydrophilicity [53]. The number of K segments in different DHN proteins is variable: twice in *CaLEA38* and *CaLEA73* and once in *CaLEA12*, *CaLEA18*, *CaLEA19*, *CaLEA11*, and *CaLEA78*. Furthermore, the Y segments (motif 3) and S segments, a serine (S)‐rich motif (motif 16), were observed only in the seven DHNs mentioned above.

### Promoter cis‐element analysis of CaLEA genes

We obtained a 2‐kb upstream region of the translation initiation sites for all *CaLEA* genes from the pepper genome database in order to investigate promoter cis‐elements among *CaLEAs* (Fig. S1). Among the subfamilies, the promoter of LEA_2 contains the highest number of cis‐elements, followed by DHN, LEA_1, and LEA_3. The promoters of *CaLEAs* encompass numerous cis‐acting regulatory elements involved in defense and stress responsiveness, including TC‐rich repeats, DRE, LTR, and MBS. This provides strong evidence that *LEA* genes are involved in responding to environmental stress. Furthermore, some promoters of *CaLEAs* also possess cis‐acting regulatory elements associated with seed or endosperm‐specific expression, such as AACA_motif and GCN4_motif.

### Chromosome distribution, gene duplication, and synteny analysis of CaLEA genes

**Fig. 3:**
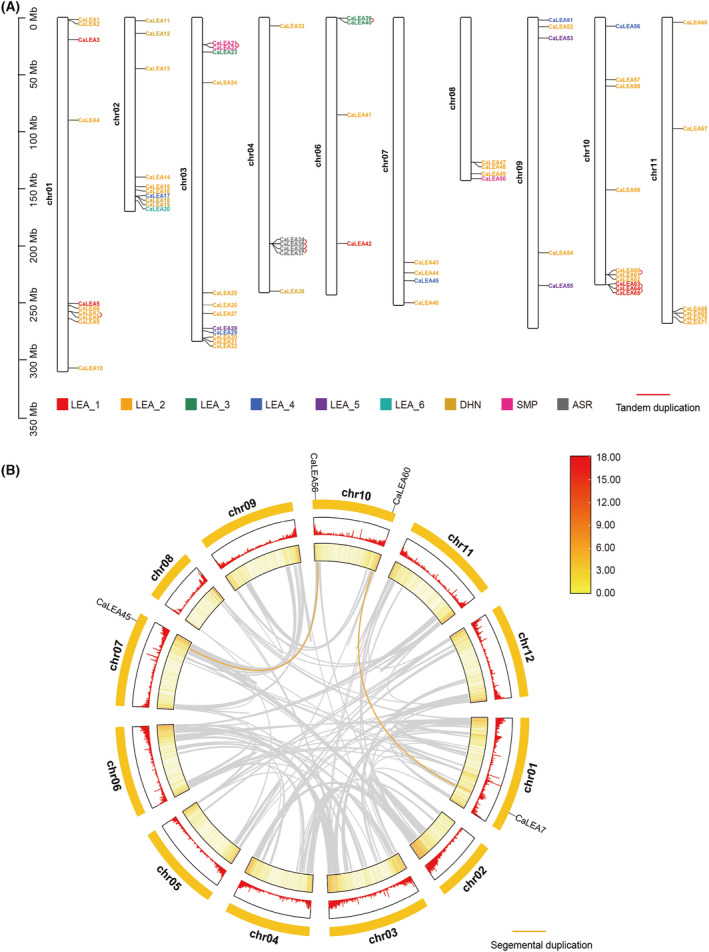
Genomic distribution of *CaLEAs* on pepper chromosomes. (A) Distribution of *CaLEA* genes on pepper chromosomes. The nine main groups are marked with differently colored fonts. The red lines indicate tandem duplication genes. (B) Synteny analysis of 71 *CaLEA*s. The rectangle on the outer ring represents pepper chromosome 01–12. The orange lines on chromosomes represent segmental duplication gene pairs.

We then mapped the chromosomal location of each *CaLEA* gene to better understand the relationship between genetic divergence, gene duplication, and the loss of the LEA family in *C. annuum* (Fig. 3A). *C. annuum* possesses 12 chromosomes, and 72 *CaLEA* genes are distributed on 10 chromosomes except chromosome 05. Because of incomplete genome sequences of *C. annuum*, the remaining 11 genes were distributed on unknown chromosomes because of incomplete genome sequences of *C. annuum*. *CaLEA* genes were also unevenly distributed among the chromosomes. As shown in Fig. 3A, 12 genes were localized on chromosome 03, whereas only four genes were localized on chromosome 06, possibly because of differences in chromosome size and structure. In addition, nine subfamilies were unevenly distributed in the genome. Members of the LEA_2 subfamily are widely distributed across 10 chromosomes, whereas other LEA subfamilies are mainly distributed on specific chromosomes, such as *ASR*, which is present only on chromosome 04. This suggests that these genes tend to replicate and evolve more conservatively within the chromosomes.

Gene duplication also played an important role in the evolution of gene families. We found two pairs of segmental duplication and eight pairs of tandem duplications in the CaLEA gene family (Fig. 3B). Eight tandem duplication pairs were located on chromosomes 01, 04, 06, and 10, which belonged to LEA_1, LEA_2, ASR, and SMP. Interestingly, all ASRs are tandem duplication genes. The two segmental duplication pairs were *CaLEA45*/*CaLEA56* and *CaLEA7*/*CaLEA60*. *K*_a_/*K*_s_ ratios were calculated for the replicated CaLEA gene pairs in pepper (Table S3). The results showed that the *K*_a_/*K*_s_ ratios of all gene pairs were < 1, suggesting that *CaLEA* genes probably underwent a strict purifying selection effect during the evolutionary search. This also implies that the duplication genes are structurally stable, evolutionarily conserved and likely to have consistent functions. The divergence time refers to the approximate date of a duplication event. Eight tandem duplication events occurred 10–115 million years ago (MYA), and the divergence times of the two segmental duplication events were 45 and 149 MYA.

We also conducted synteny analysis between pepper and other species, including *Arabidopsis*, potato, and tomato (Fig. S2). We identified a total of 29 pairs of homologous genes between pepper and *Arabidopsis*, 57 pairs between pepper and tomato, and 62 pairs between pepper and potato (Table S4). Remarkably, there is a higher number of *LEA* homologous gene pairs between pepper and the two other Solanaceae species, tomato and potato, indicating a high level of evolutionary conservation among *LEA* genes in these species. Additionally, we found that 44 *CaLEA* genes share one or two homologous gene pairs in tomato and potato, such as *CaLEA22* and *CaLEA60*, suggesting their significant role in the evolution of Solanaceae. Some of these *CaLEA* genes also have homologous pairs in *Arabidopsis*, tomato, and potato.

### Expression profiles of CaLEA genes in different tissues and organs

**Fig. 4:**
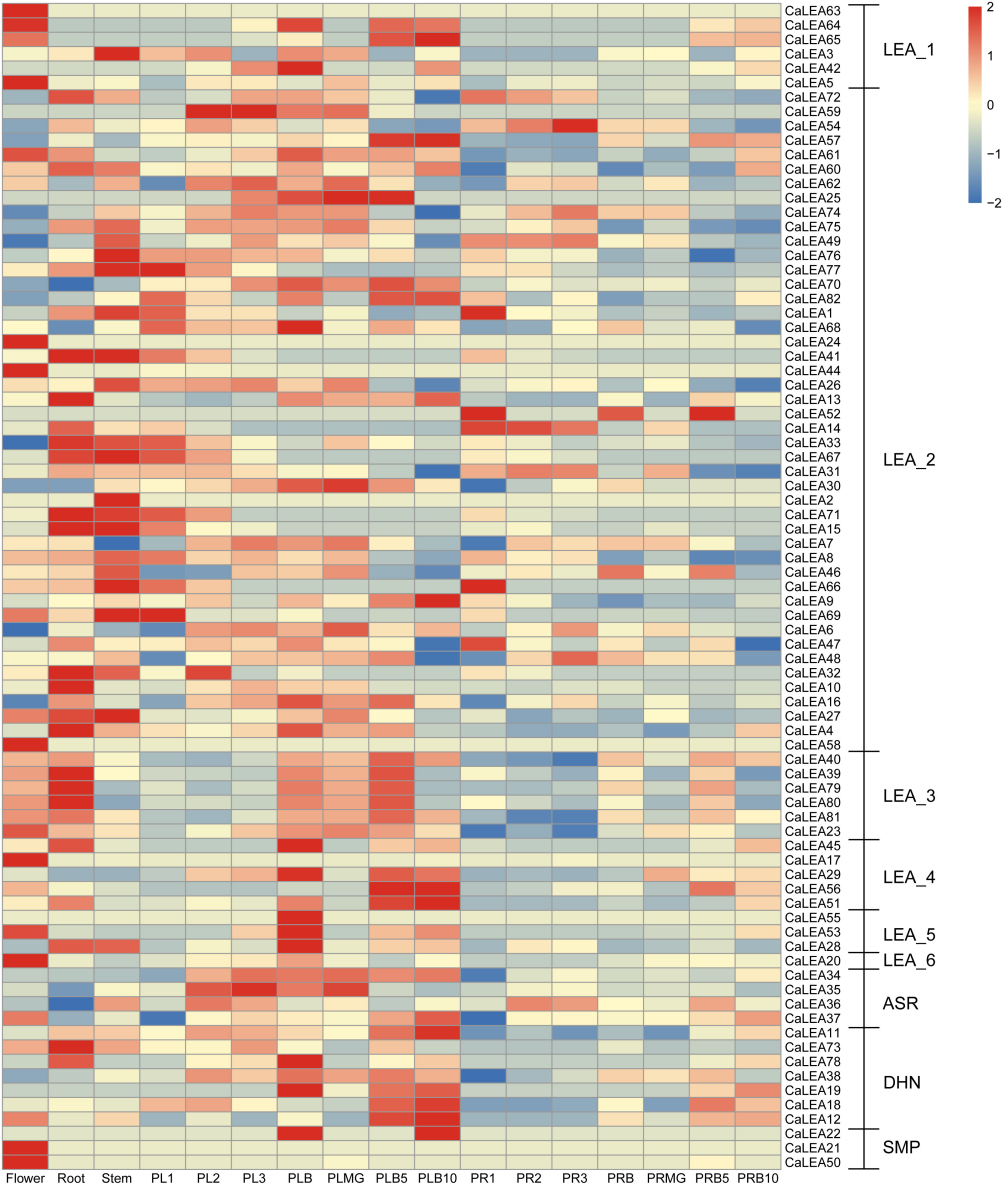
Expression profiles of *CaLEA* genes in different tissues of pepper. Dynamic expression profiles of *CaLEA* genes for 15 different development tissues or organ systems according to publicly available transcriptome data. Seven periods (1, 2, 3, mature green, breaker, breaker plus 5 days, breaker plus 10 days) of placenta and pericarp were included in the data.

To investigate the expression patterns of *CaLEA* genes in different tissues and organs of pepper plants, systematic analyses of publicly available transcriptome data were performed. Most *CaLEA* genes exhibited diverse tissue‐specific expression patterns across 17 tissues of pepper, indicating that they are likely to be engaged in diverse developmental processes. The placenta is the part of the plant where the ovules grow. Overall, the expression levels of *CaLEAs* in the placenta were higher than those in the pericarp, indicating that most *CaLEAs* play a crucial role in ovule development, particularly during the fruit‐breaking period (Fig. 4). Notably, *CaLEA20*, *CaLEA5*, *CaLEA50*, *CaLEA44*, *CaLEA17*, *CaLEA21*, *CaLEA63*, *CaLEA24*, and *CaLEA58* from five different subfamilies were highly expressed in flowers, suggesting they may participate in flower development. The mRNA of most members of the LEA_2 group, such as *CaLEA15*, *CaLEA67*, and *CaLEA41*, accumulated in the stems and roots, indicating their involvement in the development of these tissues or organs. Most duplicated gene pairs had relatively similar expression patterns (*CaLEA7*/*CaLEA8*, CaLEA39/*CaLEA40*, *CaLEA64*/*CaLEA65*, *CaLEA45*/CaLEA56, and *CaLEA34*/*CaLEA35*/*CaLEA36*/*CaLEA37*), although the expression values were different in a single tissue. However, two genes (*CaLEA43* and *CaLEA58*) were silenced in all tested tissues.

### Expression profiles of CaLEAs under abiotic stresses

**Fig. 5:**
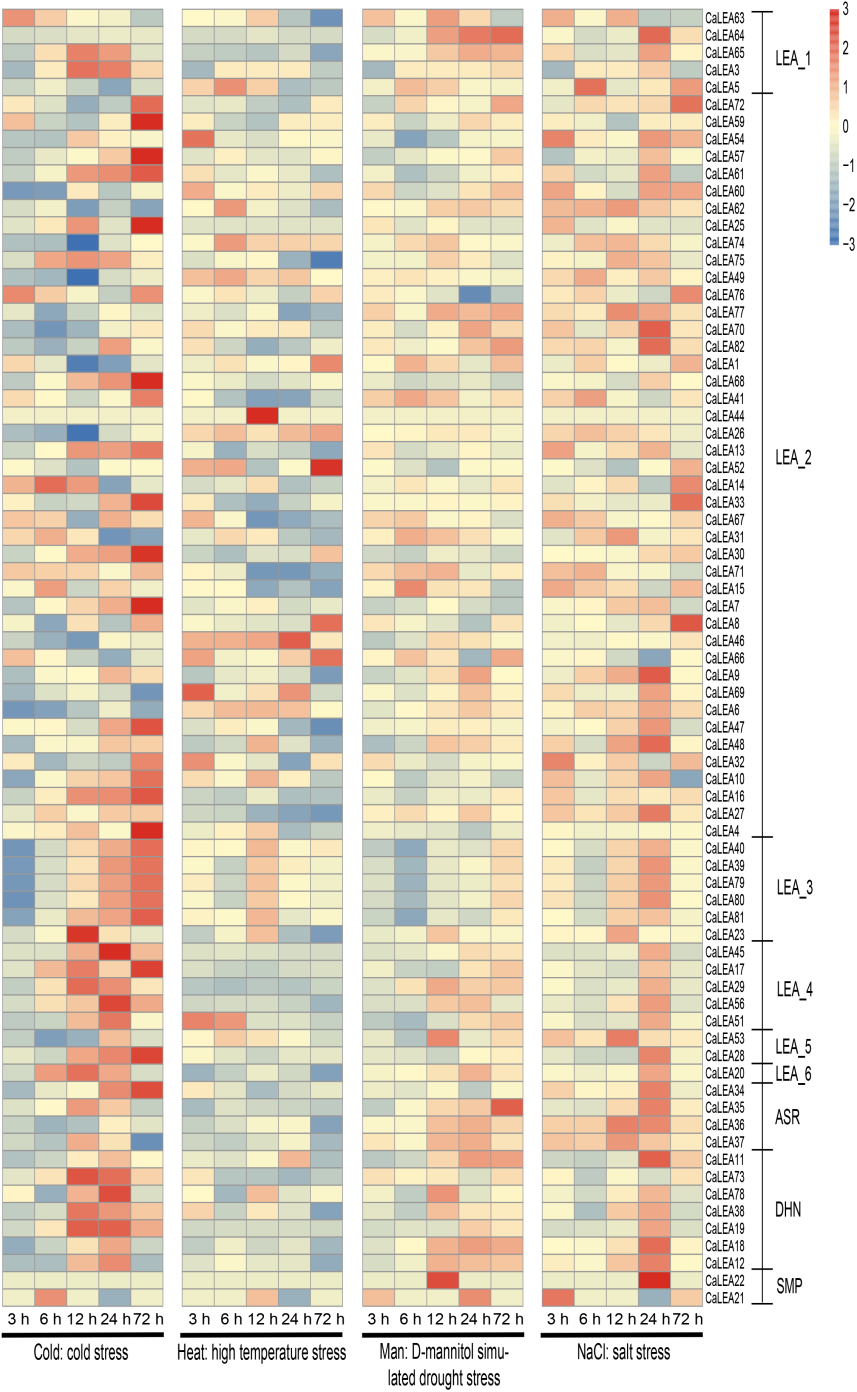
Expression changes of *CaLEA* genes in pepper under different abiotic stress treatment in pepper. 3 h, 6 h, 12 h, 24 h, and 72 h represent the fold changes after different stress treatments at 3 h, 6 h, 12 h, 24 h, and 72 h treatments, respectively.

**Fig. 6:**
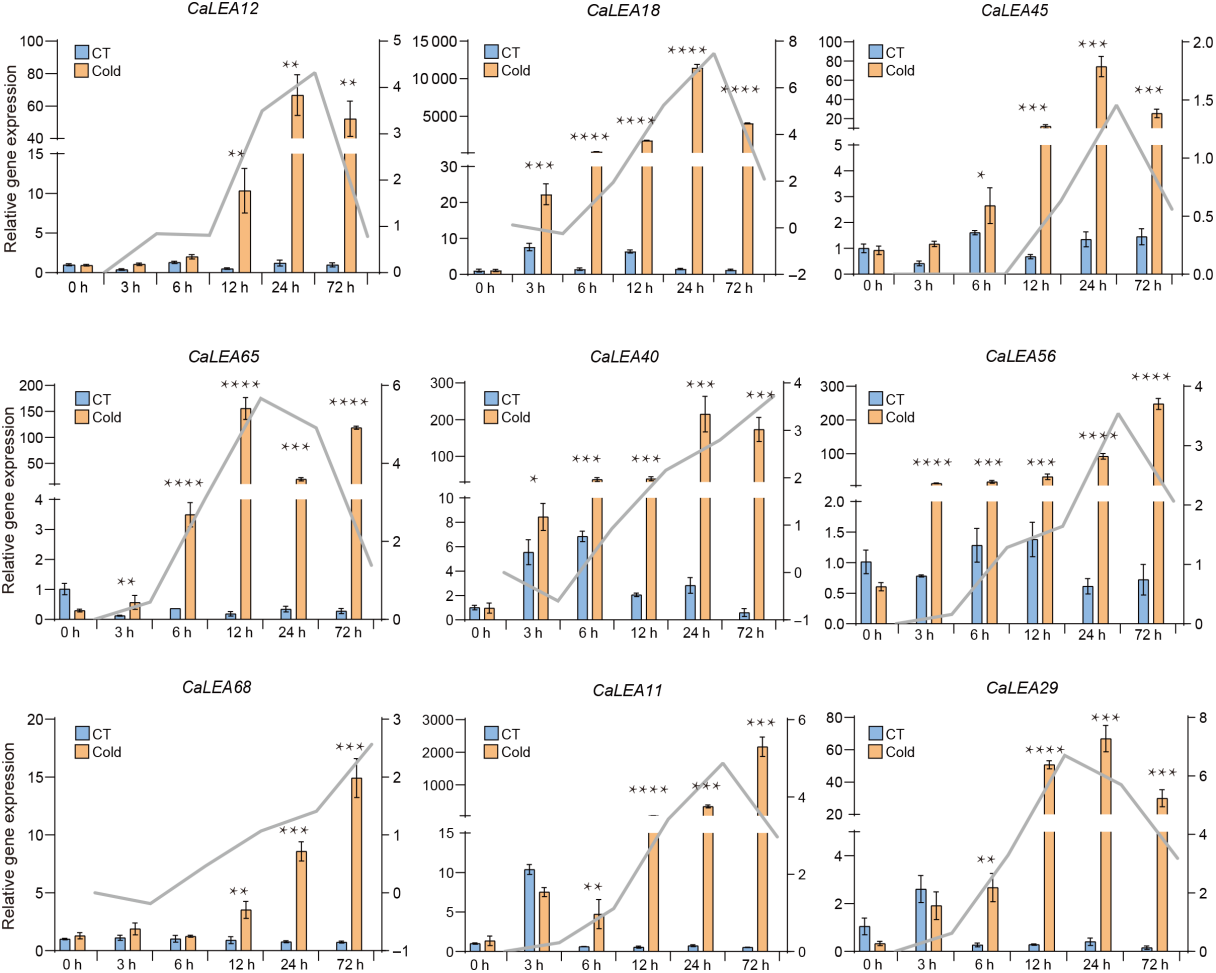
qRT‐PCR analysis of the selected *CaLEA* gene expression under cold stress. Pepper leaves were picked up at 0, 3, 6, 12, 24, and 72 h after 10 °C cold treatments. Data represent the mean ± SD of three independent experiments. The gray lines are the result of trends in the transcriptome data. Error bars indicate standard deviations (SD, *n* = 3) of independent biological replicates. An asterisk on the bars indicates a significant difference by *t*‐test. ****P ≤ 0.0001, ***P ≤ 0.001, **P ≤ 0.01, *P ≤ 0.05 in *t*‐test.

Previous studies that *LEA* genes are induced when plants are exposed to stress such as drought, low temperatures, and osmotic stress [34]. Therefore, the expression profiles of *CaLEA* genes were examined under abiotic stress responses, including cold, heat, salt, and osmotic stresses, using publicly available transcriptome data (Fig. 5). In addition to *CaLEA2*, *CaLEA24*, *CaLEA37*, *CaLEA43*, *CaLEA50*, *CaLEA55*, and *CaLEA58*, the remaining *CaLEA* genes were responsive to at least one stress. It was found that about 3/5 of *CaLEA* genes were upregulated during 6–72 h of cold stress. *CaLEA18*, *CaLEA65*, *CaLEA40*, *CaLEA56*, *CaLEA12*, *CaLEA45*, *CaLEA11*, and *CaLEA29* exhibited robust responses to cold stress, which were further confirmed through validation using quantitative reverse transcription polymerase chain reaction (qRT‐PCR; Fig. 6). The expression of these nine genes was rapidly upregulated after cold treatment and accumulated to a maximum after 12–72 h. Among them, *CaLEA68* and *CaLEA40* peaked at 72 h, while *CaLEA12*, *CaLEA18*, *CaLEA45*, *CaLEA11*, and *CaLEA56* all peaked at 24 h. MicroRNAs are important regulators of gene expression. Therefore, predictive analyses were conducted between nine *CaLEAs* in Fig. 6 and miRNAs (Table S6). The prediction results showed that the gene expression of *CaLEA68*, *CaLEA40*, and *CaLEA45* might be regulated by miRNAs. Specifically, *CaLEA68* may be regulated by three miRNAs, including can‐miRC21‐3p, can‐miR1446a‐5p.2, and can‐miR1446b‐5p.2. We also found that the mRNA levels of most *CaLEA* genes increased after 24 h of salt stress, especially *CaLEA22*. Simultaneously, some LEA_1, LEA_2, and ASR genes were repressed under heat stress, whereas other subfamilies did not significantly respond to heat stress. Another promising finding was that most LEA_1, LEA_3, LEA_4, LEA_5, LEA_6, and DHN were induced under drought stress from 12 to 72 h.

### Effect of exogenous hormone treatment on CaLEA expression

**Fig. 7:**
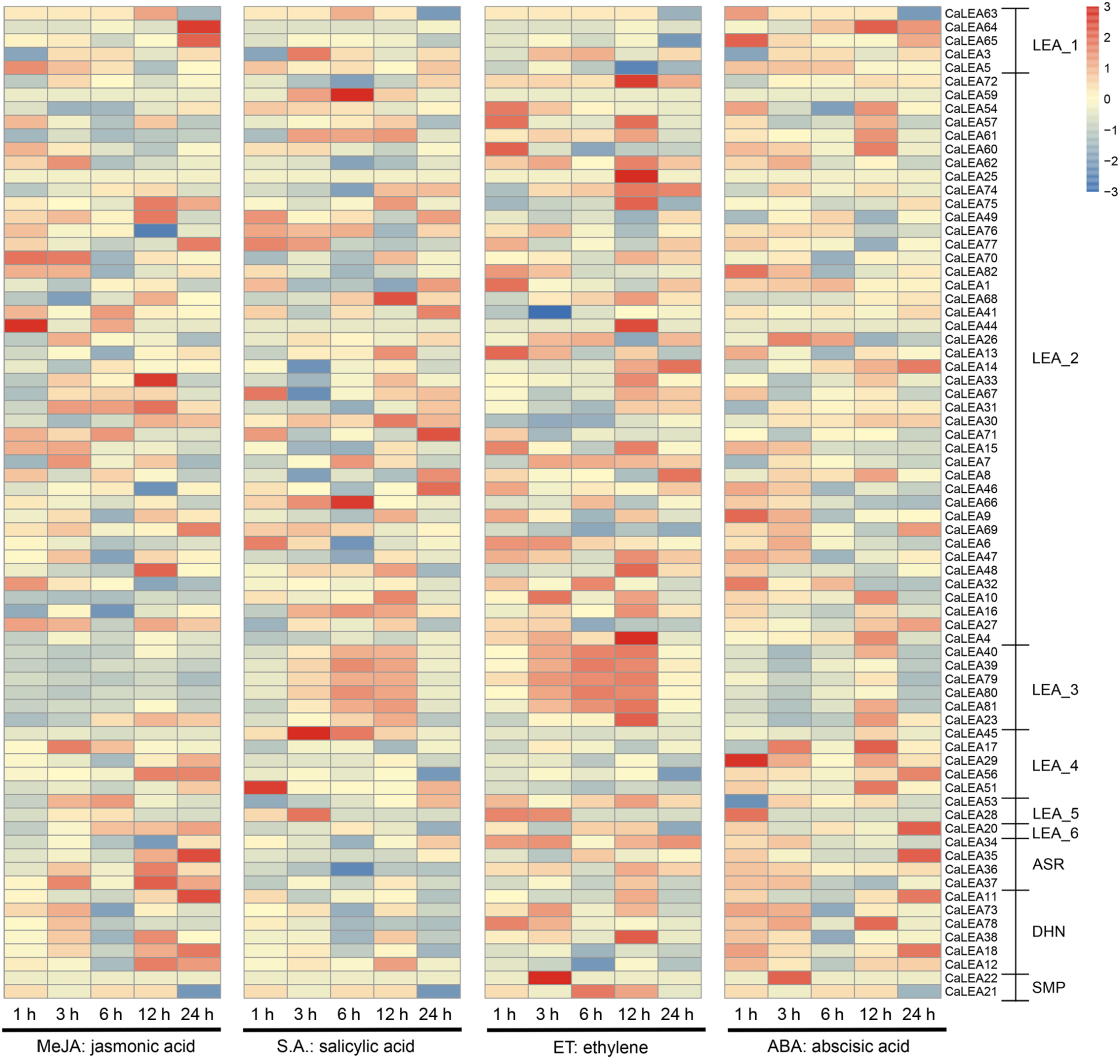
Expression changes of *CaLEA* genes in pepper under different phytohormone treatments. 1, 3, 6, 12, and 24 h represent the fold changes after different stress treatments at 1, 3, 6, 12, and 24 h, respectively.

To determine whether hormones induce the expression of *CaLEAs*, we investigated the responses of *CaLEAs* to four hormones (MeJA, methyl jasmonate; SA, salicylic acid; ET, ethylene; and ABA, abscisic acid) using transcriptome data (Fig. 7). Most LEA_3 genes were significantly upregulated after SA and ET. Some genes showed significant changes in expression levels after 24 h of either ABA or MeJA treatment, such as *CaLEA35*, *CaLEA20*, *CaLEA56*, *CaLEA11*, and *CaLEA20* were upregulated, while *CaLEA8* and *CaLEA1* were downregulated (Fig. 7). In addition, extremely different expression patterns were observed in several replicated gene pairs. By way of illustration, the maximum accumulation of *CaLEA45* occurred 3–6 h after SA treatment, but the expression level of *CaLEA*56 decreased a little after SA treatment.

## Discussion

Late embryogenesis abundant belongs to a class of proteins that participate in embryonic development and abiotic stress responses. Genome‐wide analyses of *LEA* genes have been conducted in several species. In this study, we identified 82 *LEA* genes in the pepper (*C. annuum* cv. CM334) genome, which were divided into nine subfamilies. The LEA_2 subfamily contained the most genes, accounting for 41% of the CaLEA family. This proportion is similar to that observed in most of the species studied thus far [25, 26, 30, 54, 55]. LEA_6 is the smallest subfamily with only one member, but LEA_6 subfamily is absent in a few species, such as tomato, tea plants, and *Ramonda serbica* Panc. [13, 30, 36]. The number of *CaLEA* genes is roughly equal to that of other Solanaceae species such as tobacco [55] and potato [26], and the distribution of the number of subfamily genes is also similar. In addition, the AtM subfamily was not detected in pepper, probably because it is only present in the Cruciferae [9]. These results suggest that there are variations in the LEA gene family in certain plants. Based on our findings, all CaLEA family proteins are hydrophilic, except for 13 members of the LEA_2 subfamily. This result is consistent with those of previous studies on *S. tuberosum* [26] and *A. thaliana* [9]. The members of the LEA_2 subfamily have been reported to exhibit distinct characteristics, unique structures, and potentially novel functions. This could be attributed to the fact that genes in the LEA_2 subfamily are heterologous with other LEA subfamilies. Structurally, LEA_2 subfamily proteins are highly hydrophobic, which can be attributed to the presence of the Water stress and Hypersensitive response (WHy) domain in these proteins. Furthermore, it has been observed that some members of the LEA_2 subfamily possess transmembrane helices [56]. There are two pairs of tandem duplication and one pair of segmental duplication belongs to LEA_2, which supports the previous study that duplication contributed to the expansion and diversity of LEA_2 [57].

Analysis of motifs revealed significant differences among different subfamilies, while members within the same subfamily frequently shared similar motifs. This indicates that these conserved motifs play an important role in the evolution of the LEA gene family. Previous studies have reported that LEA proteins may require specific motifs for performing their specific functions to recognize and bind specific ligands. For instance, LEA_5 and LEA_3 groups each owned one specific motif, motif 14, and motif 2. Because of their amino acid composition, LEA proteins are disordered along their sequence [58]. For example, a group of desiccation‐upregulated LEA proteins in *R. serbica* Panc., particularly LEA_1, LEA_3, DHN proteins, showed the highest propensity for disorder, while members of LEA_2 family showed the lowest disorder propensity [30]. The structural flexibility of LEA proteins is responsible for their interactions with other macromolecules (e.g., membrane proteins), thereby protecting them from stress factors [59]. Under drought stress conditions, DHNs have been suggested to have the ability to bind DNA and chelate metals. On the contrary, other intrinsically disordered LEA proteins may play a role in adopting an amphipathic α‐helical conformation or forming intracellular proteinaceous condensates. These processes could contribute to maintaining cell membrane stability in response to drought stress [30]. The support provided by these proteins to the membranes is achieved through specific structural features. The hydrophobic strip present on the class A α‐helices is positioned in a way that it can interact with the hydrophobic fatty acid tails of the outer plasma membrane, inner mitochondrial membrane, or peroxisomal membrane. Additionally, the positive strip on these α‐helices can form electrostatic interactions with the negatively charged phosphate groups of phospholipids, further contributing to the support and stability of the membranes [30, 60]. According to a previous study, genetic evolution was the reason for the low number of introns. Intron reduction shortens the time from transcription to translation, thus making genes rapidly to produce functional proteins in response to stress [25, 61, 62]. The feature of low intron number was also found in other stress‐related gene families, such as hsp20 [63]. In this study, up to 85.4% of the *CaLEA* genes contained no intron or only one intron, which provides further evidence that *CaLEA* is a stress‐response family. Gene duplication is a leading factor in the amplification and evolution of gene families [64]. Two pairs of segmental duplications and eight pairs of tandem duplications were identified in the CaLEA family. Interestingly, most members of the ASRs and SMPs were tandemly duplicated genes, suggesting that tandem replication is the main pathway of amplification in the subfamily.

Typically, further insight into gene function can be obtained from gene expression analysis. Most *LEA* genes were prominently expressed in the placenta compared with those in the pericarp, reflecting the *CaLEA* closely associated with seed development [65]. The expression patterns of most *CaLEA* duplicated gene pairs are relatively similar, suggesting these gene pairs are evolutionarily conserved and may have uniform functions. However, a few duplicated genes showed an obvious divergence in the expression pattern, indicating they may function differently in pepper, which may be the results of evolutionary selection. Previous studies have suggested that LEA proteins are involved in abiotic stresses, including cold, drought, heat, and salt stress [25, 26, 28, 33, 35, 36, 40, 41, 66]. 3/5 *CaLEA* genes were induced under cold stress, most of which belonged to the LEA_3, LEA_4, DHN, and LEA_2 subfamilies, whereas 1/5 genes were significantly repressed. Furthermore, gene expression in the DHN subgroup was induced under osmotic, salt, and cold stresses. Chen *et al*. [66] revealed that the expression of *CaDHN1* cloned from *C. annuum* P70 was significantly upregulated following treatment with osmotic stress, salt stress, cold stress, and SA. Sequence similarity between *CaLEA38* and *CaDHN1* in the DHN subgroup was 97%. *CaLEA82*, belonging to LEA_2 subfamily, is highly similar to the previously named *CaLEA6* with a sequence of more than 200 bp. In transcriptome analysis, the expression of *CaLEA82* gene increased significantly under drought and salt stress, but high temperature and low temperature did not affect the expression. This result supports the function of *CaLEA6*, which was previously studied to play a potentially protective role when dehydration and high salinity caused water deficit [39]. Lim *et al*. discover that *CaDIL1* positively regulates the ABA signaling and drought stress. *CaDIL1* corresponds to *CaLEA29* in our study, and the transcriptome analysis can also prove the correctness of previous studies [67]. Similar results have been reported in previous studies; LEA_1, LEA_2, LEA_4, and DHN subfamily genes were responsive to salt and drought stress in tomato, and some abiotic stress‐responsive cis‐elements are also found in the upstream sequences of most *SlLEA*s [36]. In *Sa. miltiorrhiza*, the majority of *SmLEA* genes were also induced by drought stress [35]. Specifically, this includes most members of the SmLEA_1, SmLEA_4, and SmSMP subfamilies, as well as all members of the SmLEA_3, SmLEA_5, and SmDHN subfamilies [35]. It is clear that the expression of *CaLEA* is induced by various abiotic stresses, suggesting that they may regulate plant responses to diverse abiotic stress‐related biological processes.

## Conclusions

In the present study, a genome‐wide analysis, including phylogenetic relationships, gene structure, gene duplication, chromosomal location, synteny analysis, and expression profiling of *LEA* genes, was performed in *C. annuum*. We identified 82 *LEA* genes in the *C. annuum* genome, and these genes were divided into nine subfamilies. Physiochemical properties and motif analyses showed that genes of the same subfamily were similar and conserved, whereas the differences between different subfamilies were very large, reflecting their similar/different functions. We also found that tandem duplication was the main factor in the expansion of the ASR and SMP subfamilies. Because *CaLEA* expression in the placenta was higher than that in the pericarp, it can be concluded that *CaLEA* is involved in pepper seed development. From the expression profile analysis, we can infer that most *CaLEAs* play important roles in abiotic stress. Overall, our results offer meaningful information for future functional studies on *CaLEA* genes.

## Conflict of interest

The authors declare no conflict of interest.

### Peer review

The peer review history for this article is available at https://www.webofscience.com/api/gateway/wos/peer‐review/10.1002/2211‐5463.13718.

## Author contributions

ZZ, ZL, YZ, FG, XG, YHu, and LF designed the study. ZZ, YZ, YHao, ZD, and WT performed the experiments. YZ, ZD, XW, JL, and LW analyzed the data. YZ, ZZ, ZL, and FG wrote the manuscript. All the authors contributed to the manuscript and approved the submitted version.

## Supporting information


**Fig. S1.** Distribution of major functional cis‐elements in the promoter sequences of the *CaLEA* genes.
**Fig. S2.** Synteny analyses of *CaLEA*s among *Arabidopsis thaliana*, *Capsicum annuum*, *Solanum lycopersicum*, and *Solanum tuberosum*.

**Table S1.** Characteristics of genes encoding LEA proteins in *C. annuum*.

**Table S2.** Conserved motifs identified by MEME tools in CaLEA proteins.

**Table S3.** Estimated Ka/Ks ratios and divergence times of duplicated CaLEAs.

**Table S4.** LEA homologous gene pairs between *C. annuum* and other species such as *Arabidopsis*, potato, and tomato.

**Table S5.** qRT‐PCR primer sequence used in this paper.

**Table S6.** Putative miRNAs and their potential target of LEA genes.

## Data Availability

The genome sequences of *C. annuum* were downloaded from the *C. annuum* (cultivar CM334) genome database (http://peppergenome.snu.ac.kr). The transcriptome data were downloaded from the NCBI Sequence Read Archive (SRA) database (https://www.ncbi.nlm.nih.gov/sra): gene expression in different tissues (leaves, stems, roots, placenta, and pericarp) of *C. annuum* (PRJNA223222); the plant in *C. annuum* response to abiotic stress, including heat, cold, salinity, and osmotic stress (PRJNA525913); and gene expression in *C. annuum* after hormone treatment (PRJNA634831).
