# Corrigendum to: Identification and expression analysis of LEA gene family members in pepper (*Capsicum annuum* L.) https://doi.org/10.1002/2211‐5463.13718

**DOI:** 10.1002/2211-5463.13875

**Published:** 2024-08-22

The Acknowledgements section of the paper by Zhao *et al*. [1] was incorrect. The correct Acknowledgements section is provided here.

