# Supplementary material for: Identification and expression analysis of LEA gene family members in pepper (Capsicum annuum L.)
Source: FEBS Open Bio. 2023 Nov 2;13(12):2246–62. doi: 10.1002/2211-5463.13718 (PMC10699114; doi:10.1002/2211-5463.13718)
Supplement: Supplementary file 1 — Fig. S1. Distribution of major functional cis‐elements in the promoter sequences of the CaLEA genes. Fig. S2. Synteny analyses of CaLEAs among Arabidopsis thaliana, Capsicum annuum, Solanum lycopersicum, and Solanum tuberosum. [file FEB4-13-2246-s001.docx]

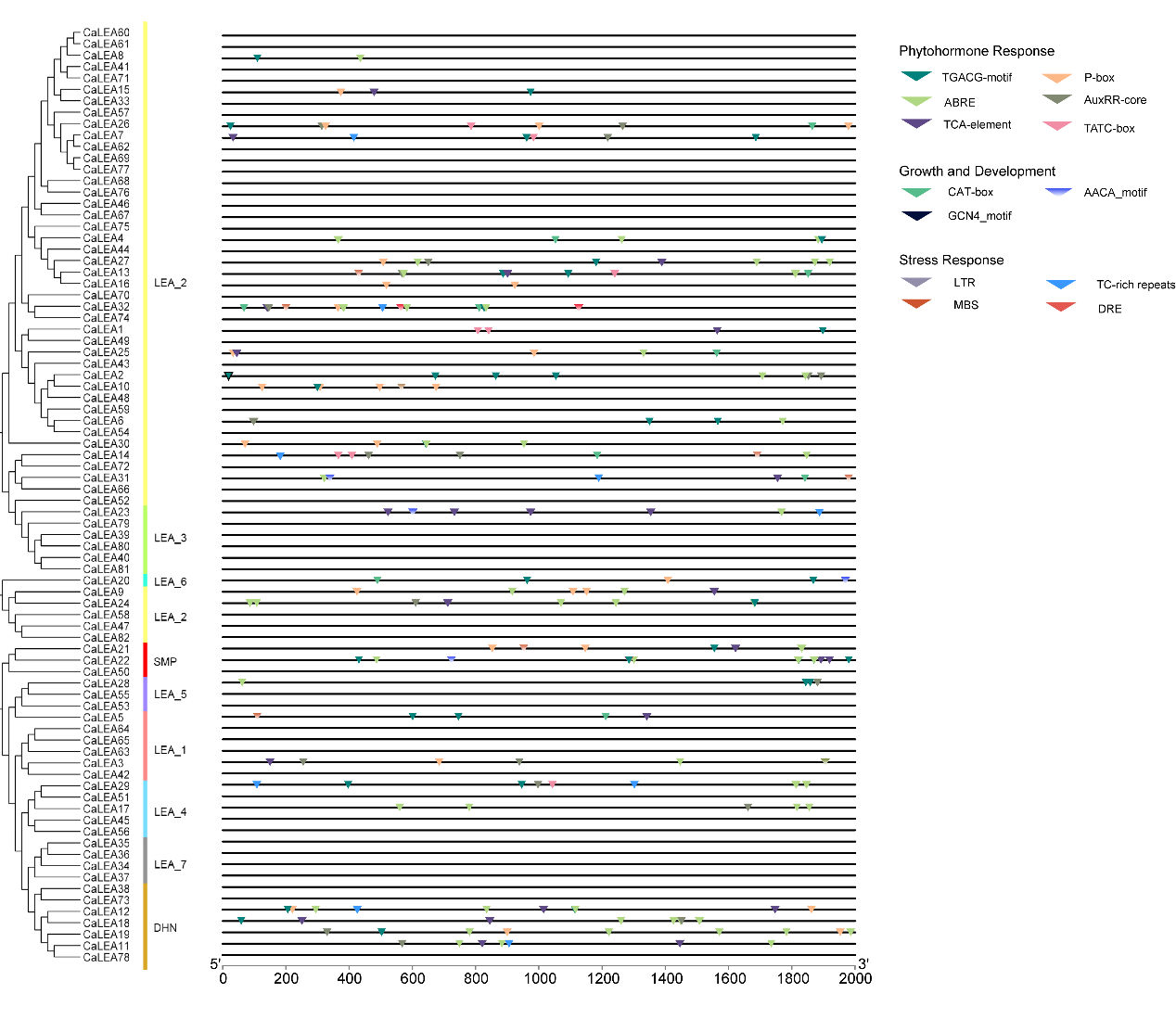


**Supplementary Figure 1.** Distribution of major functional cis-elements in the promoter sequences of the *CaLEA* genes.


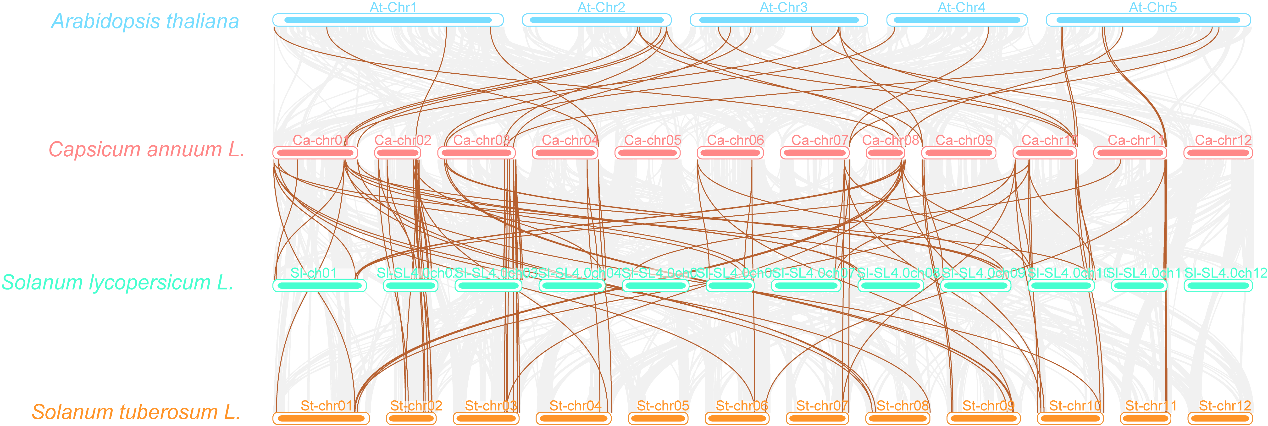


**Supplementary Figure 2.** Synteny analyses of *CaLEA*s to *Arabidopsis thaliana, Capsicum annuum, Solanum lycopersicum* and *Solanum tuberosum*. Gray lines in the background indicate collinear blocks within pepper and *Arabidopsis*, tomato, potato genomes, while brown lines highlight syntenic *LEA* gene pairs.
